# Anti–G Protein–Coupled Receptor, Class C Group 5 Member D Chimeric Antigen Receptor T Cells in Patients With Relapsed or Refractory Multiple Myeloma: A Single-Arm, Phase Ⅱ Trial

**DOI:** 10.1200/JCO.22.01824

**Published:** 2023-03-07

**Authors:** Jieyun Xia, Hujun Li, Zhiling Yan, Dian Zhou, Ying Wang, Yuekun Qi, Jiang Cao, Depeng Li, Hai Cheng, Wei Sang, Feng Zhu, Haiying Sun, Wei Chen, Kunming Qi, Dongmei Yan, Tingting Qiu, Jianlin Qiao, Ruosi Yao, Yang Liu, Xue Wang, Yanlei Zhang, Shuixiu Peng, Chih-Hua Huang, Junnian Zheng, Zhenyu Li, Alex H. Chang, Kailin Xu

**Affiliations:** ^1^ Blood Diseases Institute, Xuzhou Medical University, Xuzhou, China; ^2^ Department of Hematology, The Affiliated Hospital of Xuzhou Medical University, Xuzhou, China; ^3^ Jiangsu Key Laboratory of Bone Marrow Stem Cells, Xuzhou, China; ^4^ Shanghai YaKe Biotechnology Ltd, Shanghai, China; ^5^ Cancer Institute, Xuzhou Medical University, Xuzhou, China; ^6^ Center of Clinical Oncology, The Affiliated Hospital of Xuzhou Medical University, Xuzhou, China; ^7^ Clinical Translational Research Center, Shanghai Pulmonary Hospital, Tongji University School of Medicine, Shanghai, China

## Abstract

G protein–coupled receptor, class C group 5 member D (GPRC5D) is considered to be a promising surface target for multiple myeloma (MM) immunotherapy. Here, we report the efficacy and safety of anti-GPRC5D chimeric antigen receptor (CAR) T cells in patients with relapsed or refractory (R/R) MM.

**METHODS:**

This phase Ⅱ, single-arm study enrolled patients (18-70 years) with R/R MM. Lymphodepletion was performed before patients received 2 × 10^6^/kg anti-GPRC5D CAR T cells. The primary end point was the proportion of patients who achieved an overall response. Safety was also evaluated in eligible patients.

**RESULTS:**

From September 1, 2021, to March 23, 2022, 33 patients were infused with anti-GPRC5D CAR T cells. At a median follow-up of 5.2 months (range, 3.2‐8.9), the overall response rate was 91% (95% CI, 76 to 98; 30 of 33 patients), including 11 (33%) stringent complete responses, 10 (30%) complete responses, four (12%) very good partial responses, and five (15%) partial responses. Partial responses or better were observed in nine (100%) of nine patients with previous anti–B-cell maturation antigen (BCMA) CAR T-cell therapy, including two patients who had received repeated anti-BCMA CAR T-cell infusions with no responses at the last time. Grade 3 or higher hematologic toxicities were neutropenia (33 [100%]), anemia (17 [52%]), and thrombocytopenia (15 [45%]). Cytokine release syndrome occurred in 25 (76%) of 33 patients (all were grade 1 or 2), and neurotoxicities in three patients (one grade 2 and one grade 3 ICANSs and one grade 3 headache).

**CONCLUSION:**

Anti-GPRC5D CAR T-cell therapy showed an encouraging clinical efficacy and manageable safety profile in patients with R/R MM. For patients with MM that progressed after anti-BCMA CAR T-cell therapy or that is refractory to anti-BCMA CAR T cell, anti-GPRC5D CAR T-cell therapy might be a potential alternative option.

## INTRODUCTION

B-cell maturation antigen (BCMA) is widely expressed on the surface of mature B cells, normal and malignant plasma cells, but has low expression on other normal cells and no expression on CD34^+^ hematopoietic cells and is considered to be an ideal target for chimeric antigen receptor (CAR) T cells in the treatment of relapsed or refractory (R/R) multiple myeloma (MM).^1-5^ Studies have shown that 81%-97% of patients with R/R MM can achieve responses after infusion of anti-BCMA CAR T cells.^6-9^ However, relapses with diminished or altered surface expression of BCMA are increasingly recognized as a frequent cause of treatment failure, whereas BCMA is expressed in essentially all cases of MM at clinical presentation.^6,10^ BCMA escape could limit the potential of CAR T-cell therapy to deliver durable responses. Developing a more effective CAR T-cell regimen for additional targets may mitigate antigen loss and effectively treat patients with a low or variable BCMA expression.

**CONTEXT**

**Key Objective**
G protein–coupled receptor, class C group 5 member D (GPRC5D) is primarily expressed on malignant plasma cells in multiple myeloma (MM), and normal tissue expression is restricted to the hair follicle. We assessed the efficacy and safety of anti-GPRC5D chimeric antigen receptor (CAR) T cells in patients with relapsed or refractory (R/R) MM.
**Knowledge Generated**
Our results showed that the overall response rate was 91% in 33 patients with R/R MM, including 21 (64%) complete responses or better. No patients developed ≥ grade 3 cytokine release syndrome. The study demonstrates that anti-GPRC5D CAR T cell is another promising CAR-T therapy for patients with R/R MM.
**Relevance *(S. Lentzsch)***
Anti-GPRC5D CAR T-cell treatment is another effective CAR-T cell option, especially for R/R MM patients previously treated with anti-B-cell maturation antigen immunotherapy.*
  *Relevance section written by *JCO* Associate Editor Suzanne Lentzsch, MD, PhD.

G protein–coupled receptor, class C group 5 member D (GPRC5D), a type C 7-pass transmembrane receptor protein, is expressed on malignant and normal plasma cells at least 500-fold higher in bone marrow than that in peripheral plasma cells, and normal tissue expression is restricted to the hair follicle.^11,12^ Overexpression of GPRC5D was associated with plasma cell burden and genetic aberrations in patients with MM, and the selective expression of GPRC5D suggested that it may be a promising target antigen for CAR T-cell therapy against MM.^13^ Early preclinical studies have demonstrated that anti-GPRC5D CAR T cells have marked activity in the human MM xenograft model.^11,14^ Furthermore, anti-GPRC5D CAR T cells did not induce alopecia or cause other clinical signs of damage to the skin in murine and nonhuman primate models.^11,14^

On the basis of these findings, we conducted a phase Ⅱ clinical trial of anti-GPRC5D CAR T-cell therapy to observe the efficacy and safety in patients with R/R MM, including patients with previous anti-BCMA CAR T-cell therapy.

## METHODS

### Study Design and Patients

We conducted a single-center, single-arm, phase Ⅱ trial of anti-GPRC5D CAR T cells in patients with R/R MM from September 1, 2021, to March 23, 2022 (Chinese Clinical Trial Register: ChiCTR2100048888). The study was approved by the Ethics Committee of the Affiliated Hospital of Xuzhou Medical University. Written informed consent was obtained from each patient before study entry in accordance with the Declaration of Helsinki. Eligible patients were between age 18 and 70 years, had histologically confirmed MM, a Karnofsky Performance score of 50 points or more, and a life expectancy of more than 12 weeks without active infections, serious liver, heart, and other diseases, and met the International Myeloma Working Group diagnostic criteria for R/R MM,^15^ which is defined as a disease that progresses on salvage treatment or progresses within 60 days of the last treatment in patients who previously obtained at least a minimal response to therapy. Patients with mental or psychologic illnesses, severe allergies, or a history of severe allergies to interleukin (IL)-2 were excluded. Additional details of inclusion and exclusion criteria are provided in the protocol.

Lymphocytes were isolated from the enrolled patients using a blood cell separator, and T lymphocytes were sorted using anti-CD3 immunomagnetic beads. The antigen recognition domain of the YK-GPRC5D BB-002 vector was obtained from a human antibody phage display library. The CAR construct was composed of single-chain variable fragment, a CD8α hinge, and transmembrane domains, 4-1BB costimulatory and CD3ζ activation domains. CD3-positive T cells were transfected with lentiviral vectors for stable expression of CARs. Transduction efficiency was determined by flow cytometric analysis between days 7 and 10 and confirmed on the day of CAR T-cell infusion (Data Supplement, online only). The expression of GPRC5D was detected by flow cytometry. Lymphodepletion regimen was performed using fludarabine (30 mg/m^2^ once daily, days -5, -4, and -3) and cyclophosphamide (750 mg/m^2^ once daily, day -5). Patients were randomly assigned to receive 10 mg/m^2^ of all-trans retinoic acid (ATRA) twice daily from day -2 to day 21. On day 0, patients received a single intravenous infusion of anti-GPRC5D CAR T cells at a target dose of 2 × 10^6^/kg (Data Supplement).

### Outcomes

The primary end point was the proportion of patients achieving an overall response, including stringent complete response (sCR), complete response (CR), very good partial response (VGPR), and partial response (PR). Clinical responses were assessed according to the International Myeloma Working Group criteria.^16^ The secondary end point was safety, including the incidence and severity of cytokine release syndrome (CRS), neurotoxicities, damage to major organs (heart, liver, and kidney), and vital signs. Adverse events (AEs) were graded according to National Cancer Institute Common Terminology Criteria for AEs version 5.0.^17^ CRS was defined and graded according to American Society for Transplantation and Cellular Therapy criteria.^18^ Immune effector cell–associated neurotoxicity syndrome (ICANS) was graded by American Society for Transplantation and Cellular Therapy criteria, and other neurotoxicities were graded by National Cancer Institute Common Terminology Criteria for AEs version 5.0.

Minimal residual disease (MRD) was monitored according to the EuroFlow protocol.^19^ MRD negativity was defined as the absence of phenotypically aberrant clonal plasma cells, with a minimum sensitivity of 10^-5^ monoclonal myeloma cells in mononuclear cells. If patients had extramedullary diseases (EMD), the assessment included physical examination and imaging techniques (magnetic resonance imaging, computed tomography, and positron emission tomography-computed tomography). To assess the amplification of anti-GPRC5D CAR T cells in vivo, we designed primers for lentiviral vector to detect the proliferation of anti-GPRC5D CAR T cells in peripheral blood by quantitative polymerase chain reaction.

### Statistical Analysis

All the analyses are descriptive in nature. The sample size was based on clinical and practical consideration to enable exploratory evaluations of activity and preliminary safety.

Response rates were compared across various prespecified subgroups, including age, sex, Karnofsky Performance score, tumor burden, cytogenetic risk, GPRC5D expression at screening, and number of previous lines of therapy, especially previous anti-BCMA CAR T-cell therapy. Descriptive statistics included medians with minimum and maximum for continuous variables and counts and percentages for categorical variables. Clapper-Pearson 95% CIs and Fisher's exact test were used for categorical variables. We used the Mann-Whitney *U* test to assess prespecified exploratory associations between biomarker levels and clinical outcomes. The Wilcoxon signed-rank test was used to compare differences in monoclonal immunoglobulins and light chains levels before and after CAR T-cell infusion. The forest plots include CIs for individual groups and differences. Spearman correlations were used to assess the correlation between peak levels of GPRC5D-CARs and treatment efficiency. All *P* values were two-sided. IBM SPSS Statistics 25 software (Armonk, NY) was used for all statistical analyses.

## RESULTS

### Patients

**FIG 1.**
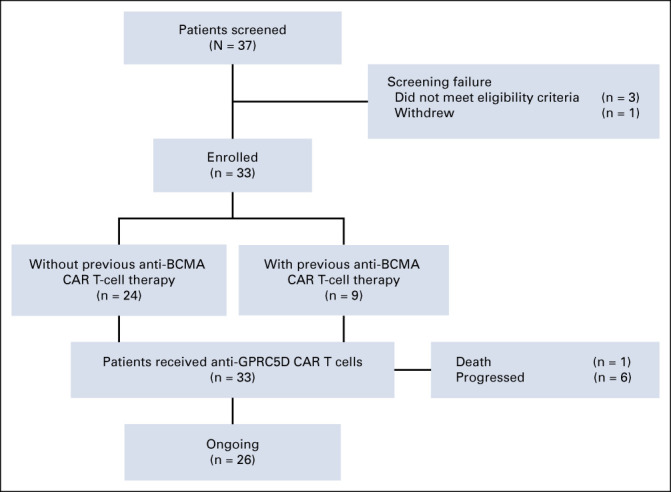
Flow diagram. BCMA, B-cell maturation antigen; CAR, chimeric antigen receptor; GPRC5D, G protein–coupled receptor, class C group 5 member D.

Between September 1, 2021, and March 23, 2022, 37 patients with R/R MM were screened and 33 were enrolled. All 33 enrolled patients received anti-GPRC5D CAR T cells and were evaluated for activity and safety (Fig 1). Patients' baseline characteristics are shown in Table 1. The median age was 58 years (range, 39-70), and 18 (55%) of 33 patients were male. The median time from MM diagnosis to anti-GPRC5D CAR T-cell infusion was 31.5 months (range, 4.8-96.0). Of 33 patients, 12 (36%) had R-ISS stage III disease, 11 (33%) had EMD, 12 (36%) had a high tumor burden, and 13 (39%) had high-risk cytogenetic abnormalities. The median of previous lines of therapy was 4 (range, 2-12). Nine (27%) patients had prior anti-BCMA CAR T-cell therapy.

**TABLE 1.**
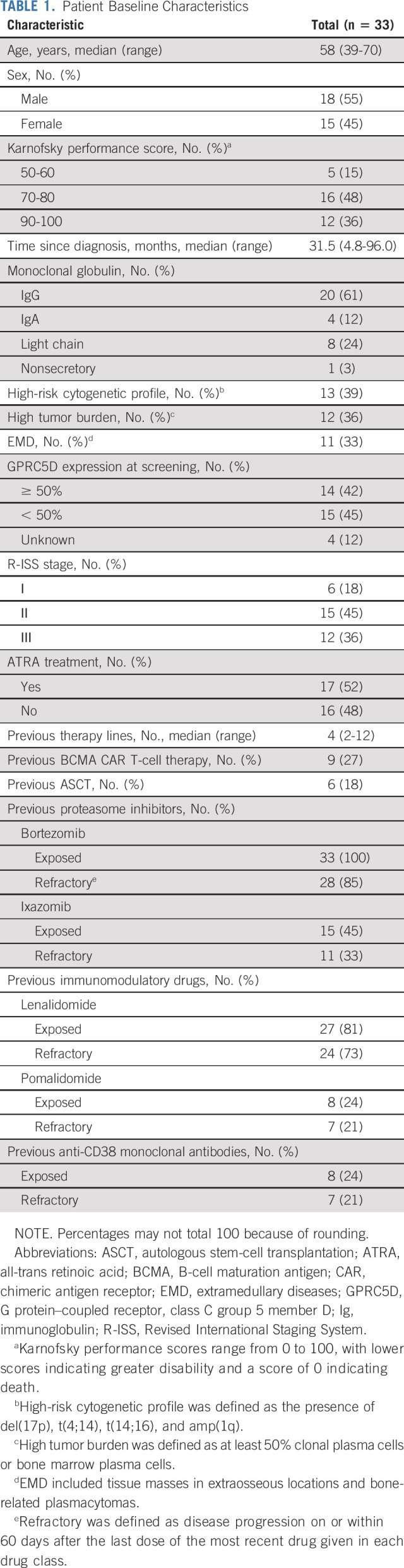
Patient Baseline Characteristics

### Efficacy

**FIG 2.**
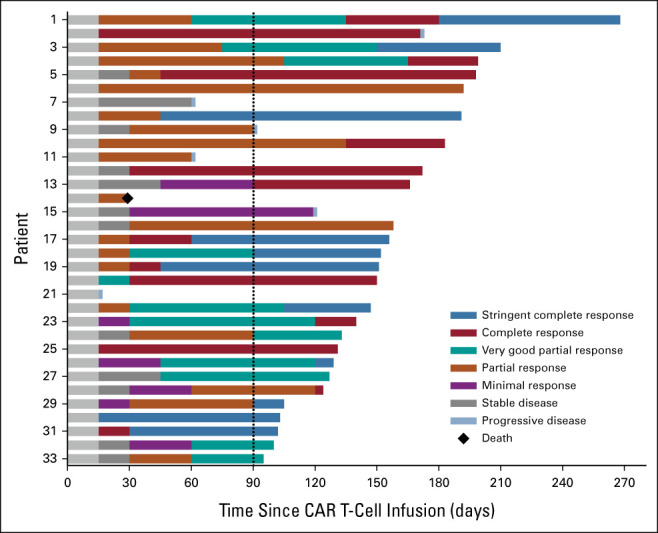
Swimmer plot of responses to anti-GPRC5D CAR T cells. The dotted line marks 3 months after anti-GPRC5D CAR-T cell infusion. CAR, chimeric antigen receptor; GPRC5D, G protein–coupled receptor, class C group 5 member D.

**FIG 3.**
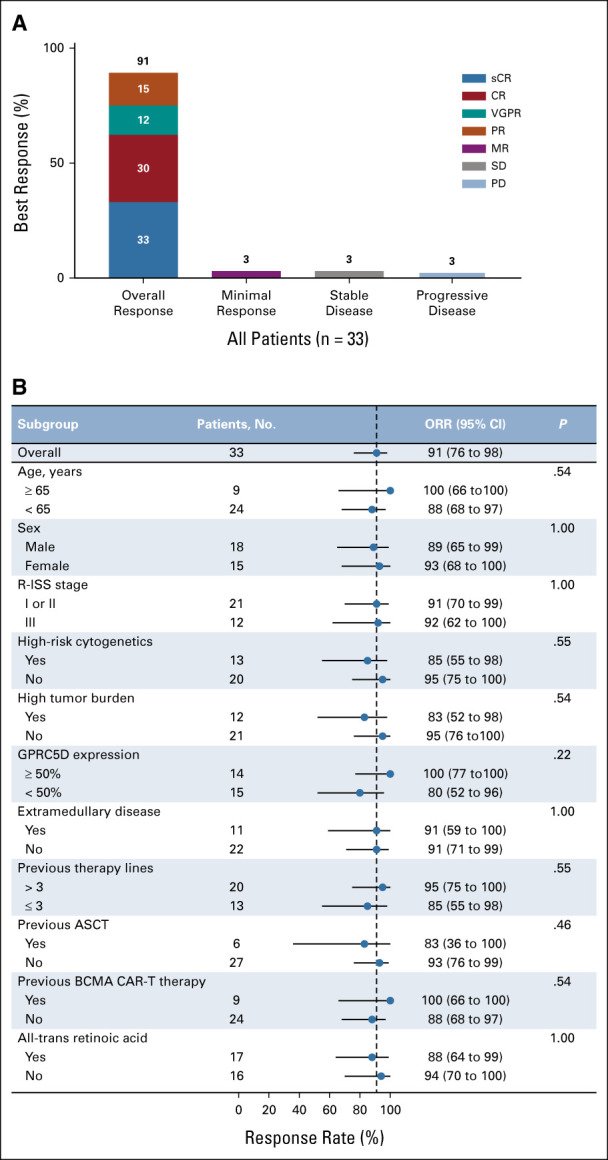
Overall responses and responses in subgroups. (A) The rates of overall response, minimal response, stable disease, and progressive disease. All responses were confirmed and assessed on the basis of the International Myeloma Working Group Uniform Response Criteria for Multiple Myeloma. Percentages may not total 100 because of rounding. (B) Response rates in subgroups. Blue dots represent the observed proportions, and the lines extending from the dots are the 95% CIs for these proportions. The 95% CI was calculated using the Clopper-Pearson method. The high-risk cytogenetic profile was defined as the presence of del(17p), t(4;14), t(14;16), and amp(1q). High tumor burden was defined as at least 50% clonal plasma cells or bone marrow plasma cells. Extramedullary diseases included tissue masses in extraosseous locations and bone-related plasmacytomas. ASCT, autologous stem-cell transplantation; BCMA, B-cell maturation antigen; CAR, chimeric antigen receptor; CR, complete response; GPRC5D, G protein–coupled receptor, class C group 5 member D; MR, minimal response; ORR, overall response rate; PD, progressive disease; PR, partial response; R-ISS, Revised International Staging System; sCR, stringent complete response; SD, stable disease; VGPR, very good partial response.

At the cutoff date (June 25, 2022), the median follow-up was 5.2 months (range, 3.2-8.9). The overall response rate was 91% (95% CI, 76 to 98; 30 of 33 patients), including 11 (33%) sCRs, 10 (30%) CRs, 4 (12%) VGPRs, and 5 (15%) PRs (Figs 2 and 3A). The median time to first responses was 0.5 month (range, 0.5-3.0), and the median time to best response was 1.8 months (range, 0.5-6.0). Disease progression occurred in three (10%) of 30 patients who had responses (Fig 2), and only one had progressive disease (PD) 171 days after anti-GPRC5D CAR T-cell infusion in 21 patients with CR or sCR. Compared with baseline, the concentrations of monoclonal immunoglobulin G, kappa, and lambda light chains in the peripheral blood of patients with responses significantly decreased within one month after treatment (Data Supplement).

Responses to anti-GPRC5D CAR T cells were observed in 14 (100%) of 14 patients with 50% or higher GPRC5D expression and 12 (80%) of 15 patients with lower than 50% GPRC5D expression (*P* = .22; Fig 3B). There was no significant difference in response rates between patients with ATRA treatment and those without exposure to ATRA. Univariate analyses showed that response rates were consistent across the other covariates (Fig 3B). Expression of GPRC5D on CD138^+^ cells by flow cytometry is shown in the Data Supplement.

Of all 33 patients, 26 patients (79%; 95% CI, 62 to 91) achieved bone marrow MRD negativity, including 11 with sCR and nine with CR. Twenty-five (96%) of 26 patients with bone marrow MRD negativity had no recorded progression events, and one (14%) of seven patients with sustained bone marrow MRD positivity had no disease progression during follow-up (Data Supplement).

### Efficacy in Patients With Previous BCMA CAR T-Cell Therapy

Nine patients underwent anti-BCMA CAR T-cell therapy before entering this clinical trial, including six patients with relapsed MM and three with PD after anti-BCMA CAR T-cell treatment (Table 2). Responses to anti-GPRC5D CAR T cells were observed in nine (100%) of the nine patients, including four patients achieving CR (Fig 3B, Table 2). There was no significant difference in response rates between patients with previous BCMA CAR T-cell therapy and those without. Two patients had received repeated anti-BCMA CAR T-cell infusions with no responses at the last time, of whom one patient achieved CR, and the other PR after anti-GPRC5D CAR T-cell infusion.

**TABLE 2.**
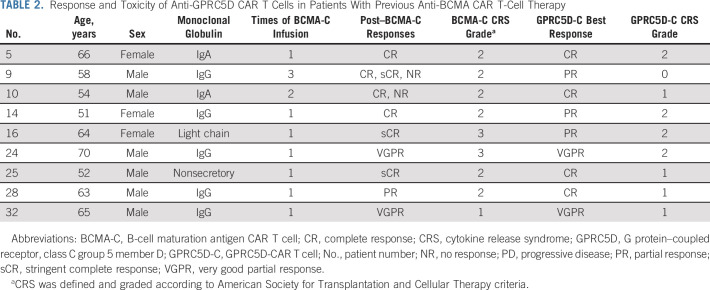
Response and Toxicity of Anti-GPRC5D CAR T Cells in Patients With Previous Anti-BCMA CAR T-Cell Therapy

### Safety

Hematologic toxicities were the most common AEs (Table 3). Grade 3 or higher hematologic AEs included neutropenia (33 [100%]), anemia (17 [52%]), and thrombocytopenia 15 (45%)]. The median duration of grade 3-4 neutropenia was 14 days (range, 2-76), that of anemia 24 days (range, 4-67), and that of thrombocytopenia 29 days (range, 5-67; Data Supplement). There were 29 (88%) patients with varying degrees of cytopenia before lymphodepleting chemotherapy, including 15 (45%) patients having neutropenia, 22 (67%) anemia, and 12 (36%) thrombocytopenia.

**TABLE 3.**
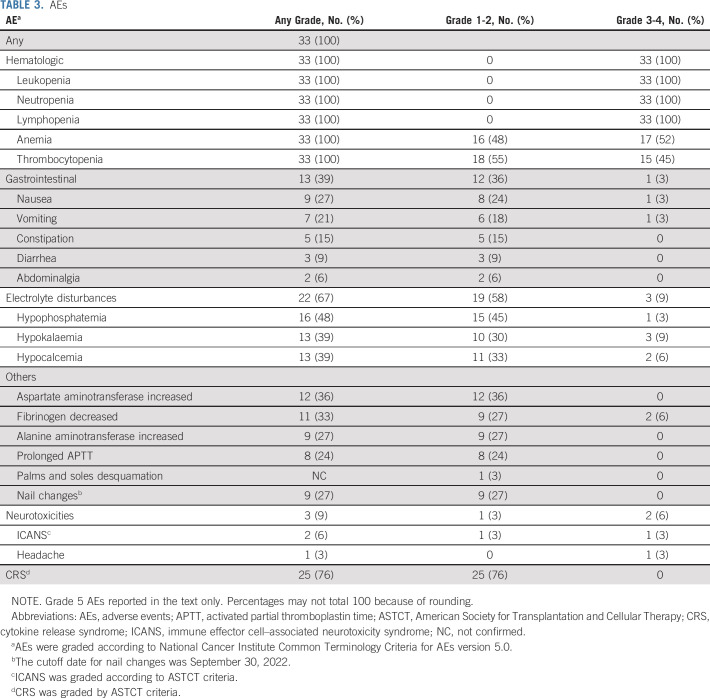
AEs

CRS was observed in 25 (76%) of 33 patients, and no grade 3 or higher CRS was observed (Table 3). The median time to onset of CRS was 7 days (range, 1-18) postinfusion, and the median duration was 3 days (range, 1-8). The levels of IL-6, ferritin, and CRP reached their median peaks on days 14, 14, and 7 after CAR T-cell infusion, respectively (Data Supplement). Patients with CRS have higher peak levels of IL-6, ferritin, and CRP than those without CRS (Data Supplement). Compared with baseline, levels of IL-6, ferritin, and CRP increased markedly during CRS (Data Supplement). All CRSs were rapidly relieved after conventional treatment including tocilizumab and glucocorticoids. Of nine patients who had received anti-BCMA CAR T-cell therapy previously, CRS occurred in eight (89%) patients after anti-GPRC5D CAR T-cell infusion, with six having lower grade of CRS than anti-BCMA CAR T cells (Table 2).

Neurotoxicity events after anti-GPRC5D CAR T-cell infusion, including ICANS and other neurotoxicities, occurred in three (9%) of 33 patients (Table 3). ICANS occurred in two (6%) patients, of whom one (3%) was grade 2 and the other was grade 3. The time to ICANS onsets was 10 and 14 days, and the duration was 3 and 5 days. Both patients obtained remission of ICANS after treatment with glucocorticoids. A grade 3 headache was observed in one patient during CRS, which was relieved after administering 300 mg oral ibuprofen twice daily and disappeared within 5 days.

Gastrointestinal events, mainly nausea, occurred in nine (27%) patients, with seven having recurrent vomiting. Five (15%) patients experienced constipation, three (9%) diarrhea, and two (6%) abdominalgia (Table 3). A grade 2 cutaneous AE, manifested as the desquamation of the palms and soles, was observed in one patient. After topical medication treatment, it disappeared within 1 week. Of the 32 patients who survived for more than 3 months, nine (28%) had grade 1 nail changes, which resolved without any intervention. The median time from anti-GPRC5D CAR T-cell infusion to nail changes was 4.0 months (range, 1.4-6.0).

One patient (patient 14) died during follow-up. The patient achieved a PR on day 14 after CAR T-cell infusion. A decreased platelet count was observed in the patient because of disease progression before CAR T-cell therapy, and the platelet counts were below the level of 20 × 10^9^/L in multiple tests throughout CAR T-cell treatment. Despite platelet transfusion, the patient died of intracranial hemorrhage 29 days after CAR T-cell infusion, and no abnormal coagulation was observed.

All the 33 patients developed B-cell aplasia. B-cell aplasia was observed in 97%, 80%, and 50% of patients at 1, 2, and 3 months, respectively (Data Supplement). Hypoimmunoglobulinemia already existed in 29 (88%) patients before anti-GPRC5D CAR T-cell infusion. Serum IgA (in patients with non-IgA MM), IgM (in patients with non-IgM MM), and IgG (in patients with non-IgG MM) decreased significantly after CAR T-cell infusion compared with baseline concentrations. Recovery of serum IgM to a normal level was observed in 40% (8 of 20) of patients after 3 months, and IgG and IgA had not recovered to the normal concentration at data cutoff, except three patients.

### CAR T-Cell Expansion and Persistence

The presence of anti-GPRC5D CAR T cells in patients' peripheral blood was evaluated by quantitative polymerase chain reaction (Data Supplement). Amplification of GPRC5D-CARs peaked between days 14 and 28 postinfusion followed by a gradual decrease. The expansion was durable, with 89% and 48% of 33 patients having detectable CAR T cells at 1 and 3 months, respectively (Data Supplement). The patients with clinical responses showed a tendency to higher peak levels than those without responses (Data Supplement), including two patients having detectable CAR T cells for more than 7 months. Peak levels of GPRC5D-CARs in patients with CRS were significantly higher than those without CRS (Data Supplement). No association of initial GPRC5D expression on myeloma cells with GPRC5D-CARs expansion was observed; also, there was no significant difference in CARs expansion between patients treated with ATRA and those without exposure to ATRA.

## DISCUSSION

Treatment with GPRC5D CAR T cells resulted in high antitumor activities in patients with R/R MM in the phase Ⅱ study. The responses were observed in 91% of the treated patients, and CRs or better were observed in 64%. The fact that MRD-negative responses occurred in approximately 80% of the patients highlights the depth of response induced by anti-GPRC5D CAR T-cell therapy. The patients were refractory to available therapies with multiple high-risk features, including 73% of patients with a high tumor burden, high-risk cytogenetic abnormalities, EMD, or failed BCMA-directed CAR T-cell therapies. Responses were observed in all these subgroups. Our study demonstrated that anti-GPRC5D CAR T-cell therapy induced encouraging efficacy in the R/R MM populations, especially in those who have failed in anti-BCMA CAR T-cell therapy or progressed after responding to the treatment.

Recently, Mailankody et al^20^ reported an impressive GPRC5D CAR T-cell study (MCARH109), which showed that 12 (71%) of 17 patients responded to MCARH109. Of 12 patients who received doses of 25 × 10^6^ to 150 × 10^6^ CAR T cells, seven (58%) had an objective response, whereas patients who received 450 × 10^6^ CAR T cells had a response rate of 100%. The response rate in our study was higher than that at doses of 25 × 10^6^ to 150 × 10^6^ CAR T cells, but lower than that at 450 × 10^6^ CAR T cells in MCARH109 therapy,^20^ indicating that dose size of anti-GPRC5D CAR T cells might lead to differences in efficacy.

BCMA-targeted CAR T-cell therapy has shown unprecedented results in treating R/R MM.^2,7,21^ However, BCMA expression on MM plasma cells is variable and heterogeneous.^6^ Membrane-bound BCMA can be actively cleaved from the tumor cell surface by γ-secretase, resulting in the reduction of surface BCMA level on MM cells, consequently resulting in a reduced anti-BCMA CAR T-cell response.^5,22^ In contrast to BCMA, GPRC5D is a type C 7-pass transmembrane receptor protein, and unlikely to be shed in the serum, leading to a sink effect and reduced efficacy for CAR T cells.^22^ Preliminary studies have shown the potential of GPRC5D for a candidate target of CAR T cell,^11-13,23-25^ and the results of MCARH109 confirmed its activity for MM in clinical trial.^20^ In our study, a high (9 of 9) response rate was observed in patients who had PDs after anti-BCMA CAR T-cell therapy. It was worth mentioning that CR and PR were obtained in two patients who had received repeated anti-BCMA CAR T-cell infusions, but had not any responses at the last time.

In this study, peak levels of GPRC5D-CARs amplification appeared to be lower in patients without clinical responses than in those with responses, but the difference was not statistically significant, and it may be related to the small number of patients without responses (n = 3). In addition, two patients with GPRC5D-CARs amplification at 7 months remained in sCR, which further supported the necessity of effective amplification of GPRC5D-CARs for clinical responses. We also analyzed the expression of GPRC5D on plasma cells before CAR T-cell therapy. There was no significant difference in response rates between the high GPRC5D expression cohort and patients with low GPRC5D expression. Previous studies have shown that ATRA induced significant amounts of GPRC5D expression,^26^ but interestingly, transcription of GPRC5D was not changed by ATRA.^27^ In our study, there was no significant difference in CAR T-cell expansion between patients with ATRA treatment and those without, which may be due to insufficient ATRA dose for safety concerns.

Therapy-related AE, CRS in particular, is an important consideration for a new CAR T-cell product, which might elicit exacerbation of conditions and treatment-related deaths. The incidence of CRS was less than 80%, and all cases were grade 1 or 2 in our study. In the study by Mailankody et al,^20^ one patient had grade 4 CRS and ICANS, which might be related to the highest dose level of MCARH109 (450 × 10^6^ CAR T cells), whereas the dose level was 2 × 10^6^/kg in our study. No ICANS of any grade or CRS of grade 3 or higher was observed in patients who received MCARH109 therapy between dose levels of 25 × 10^6^ and 150 × 10^6^ cells.^20^ The dose level in our study was just in this range, also, we did not observe CRS above grade 2 in the range, and the incidence of ICANS was low (2 patients [6%]). We noticed that the median time from CAR T-cell infusion to fever initiation was 7 days, and the median duration was 3 days, which was shorter than that previously reported for other types of CAR T cells in our center.^9,28^ All CRS events were rapidly relieved after conventional treatments, and both the patients achieved remission of ICANSs with the treatment of glucocorticoids, which demonstrated the safety of anti-GPRC5D CAR T cells.

High-grade hematologic toxic events were common but transient. Leukopenia was mainly observed after lymphodepleting chemotherapy. No severe infection occurred during the neutropenic phase, which was shortened by injection of granulocyte colony-stimulating factor. Anemia and thrombocytopenia were present in some patients before lymphodepleting chemotherapy, and further decreases were not remarkable during CAR T-cell therapy. One patient died of cerebral hemorrhage. The patient had a very low platelet count throughout treatment, but had no abnormal coagulation leading to the hemorrhage, and we presume that cerebral hemorrhage might be attributed to the severe thrombocytopenia. Because of the relatively short follow-up period, the dynamics of humoral immune reconstitution cannot be fully reflected, but IgM is the earliest to recover, similar to the anti-BCMA CAR T-cell therapy that we reported previously.^29^

Dermatologic and oral AEs have been reported in previous studies involving GPRC5D-targeted therapy in patients with R/R MM.^14,23,24,30^ In our study, the incidence of nail changes was 28%, similar to that of GPRC5D × CD3-bispecific antibody.^24^ The latest case of nail changes occurred 6 months after anti-GPRC5D CAR T-cell infusion, indicating that a longer follow-up is required to observe nail changes. Rates of other cutaneous and oral toxicities appeared to be lower than those in previous GPRC5D-targeted treatments.^20,23,30^ First of all, more than half of patients were simultaneously treated with ATRA, which may cause AEs such as cheilitis, dry mouth, paronychia, and desquamation. None of these relevant AEs were severe, so we did not list them in Table 3. Second, the races of patients were different between our and other studies, and there might be differences in AEs among different populations, which need to be confirmed with larger samples. Several gastrointestinal events were found after anti-GPRC5D CAR T-cell therapy, but they were relieved with conventional treatment. A smaller part of patients had hypophosphatemia and hypokalemia, which were controlled with appropriate and timely treatment. Obviously, the scarcity of severe toxicity showed the safe profile of anti-GPRC5D CAR T cells.

Some limitations should be considered in our study. The study population was only from China and was less heavily pretreated than some of the previous CAR T studies,^8,31^ and some patients were not exposed to IMiD or CD38 monoclonal antibody. In addition, follow-up time was relatively short, and future study will be required to assess the activity and AE of long-term.

In conclusion, anti-GPRC5D CAR T-cell therapy induced a high response rate, and the grades of all CRS events were 1 to 2, with low incidence of ICANS in the study. Anti-GPRC5D CAR T cells might be a very potential candidate treatment for patients with MM progressed after anti-BCMA CAR T-cell therapy or refractory to anti-BCMA CAR T cells.

## Data Availability

The authors declare that parts of the data supporting the findings of this study are included in the Data Supplement. The participant data underlying the results are available upon reasonable request from the corresponding author Kailin Xu.
